# Underwater endoscopic submucosal dissection of a 20-cm-long circular rectosigmoid polyp

**DOI:** 10.1055/a-2610-2567

**Published:** 2025-06-18

**Authors:** Jens Aksel Nilsen, Anders Egeland, Petter Tandberg, Ole Darre-Næss, Espen R. Norvard, Stine Hvattum

**Affiliations:** 1 155273 Department of Medicine, Bærum Hospital, Vestre Viken Hospital Trust, Gjettum, Norway; 2 155272 Department of Transplantation Medicine, Oslo University Hospital, Oslo, Norway; 3 155273 Department of Pathology, Drammen Hospital, Vestre Viken Hospital Trust, Drammen, Norway

Circular colorectal polyps present significant challenges for endoscopic resection, particularly when en bloc removal is required to increase the likelihood of curative treatment in lesions with potential malignant foci. Conventional endoscopic submucosal dissection (ESD) of such lesions is technically demanding, time-consuming, and complicated by prolonged procedure duration and marked bowel distention from CO₂ insufflation. Underwater ESD offers several advantages over conventional ESD. By minimizing bowel distension, it helps maintain stable respiratory and hemodynamic parameters during prolonged procedures. Underwater precoagulation of submucosal vessels has recently been described, saving time and improving procedural efficiency 1 . In addition, the elimination of smoke and the magnification effect of the water provide a clearer view of the submucosal space during dissection ( Fig. 1 ). Reduced colonic distension further enhances scope maneuverability in lengthy procedures 2 3 4 .

**Fig. 1:**
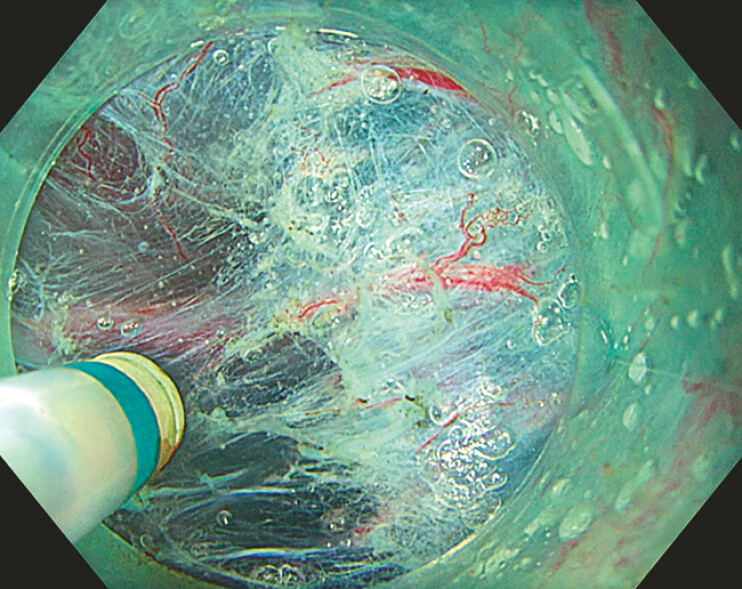
Underwater view of the submucosal space during endoscopic submucosal dissection.

We report the case of a 58-year-old man with no significant medical history who had a circular, laterally spreading tumor of the granular mixed type spanning from the lower rectum to the sigmoid colon with several large nodules (>10 mm), including one with type 2B features (Japan Narrowband Imaging Expert Team [JNET] classification). Magnetic resonance imaging excluded locoregional malignancy. En bloc resection was indicated, and ESD was performed under general anesthesia. The dissection was predominantly performed underwater with isotonic saline, which facilitated the procedure by reducing bowel distension and maintaining a stable operating field ( Video 1 ). The resection was successful, yielding a specimen measuring 22 cm by 15 cm after sectioning ( Fig. 2 , Fig. 3 ). Dissection time was 480 minutes, corresponding to a dissection speed of 54 mm ^2^ /min. Histopathology confirmed a tubulovillous adenoma with high-grade dysplasia and an R0 resection.

Underwater endoscopic submucosal dissection of a circumferential rectosigmoid polyp. The video highlights the enhanced optics and the elimination of smoke during submucosal dissection. SITE, saline-immersion therapeutic endoscopy.Video 1

**Fig. 2:**
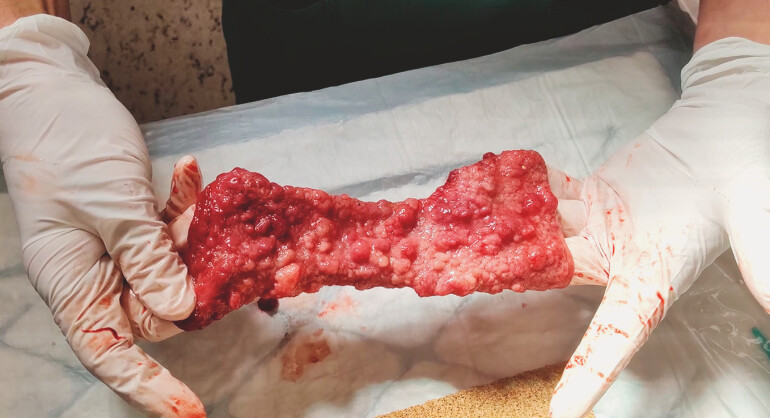
En-bloc specimen of the circular, laterally spreading tumor of the granular mixed type that spanned from the lower rectum to the sigmoid colon.

**Fig. 3:**
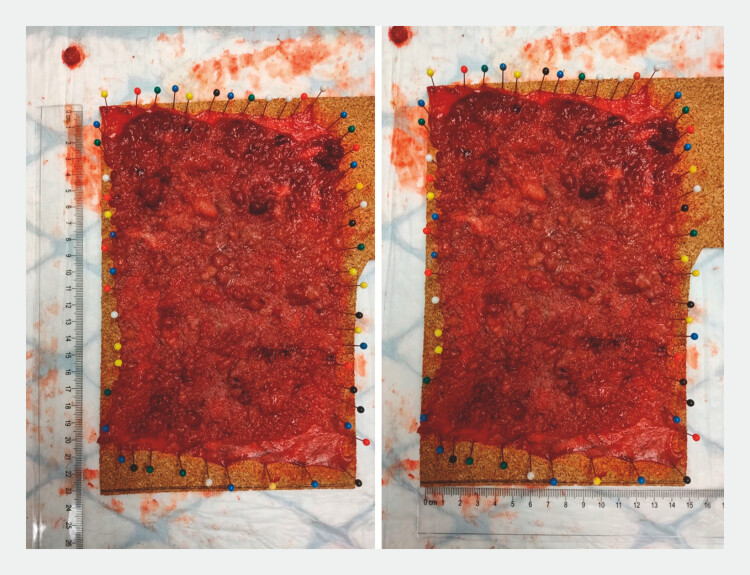
Specimen pinned to a cork plate and measuring 15 × 22 cm.

Postoperatively, the patient was treated with a course of oral prednisone (30 mg tapered by 5 mg per week). Eight weeks later, a 2-cm-long stenosis developed, which was successfully managed with balloon dilation.

Endoscopy_UCTN_Code_TTT_1AQ_2AD_3AD

## Notes

Endoscopy E-Videos https://eref.thieme.de/e-videos
*E-Videos* is an open access online section of the journal *Endoscopy* , reporting on interesting cases and new techniques in gastroenterological endoscopy. All papers include a high-quality video and are published with a Creative Commons CC-BY license. Endoscopy E-Videos qualify for HINARI discounts and waivers and eligibility is automatically checked during the submission process. We grant 100% waivers to articles whose corresponding authors are based in Group A countries and 50% waivers to those who are based in Group B countries as classified by Research4Life (see: https://www.research4life.org/access/eligibility/ ). This section has its own submission website at https://mc.manuscriptcentral.com/e-videos .
